# Extended diversity analysis of cultivated grapevine *Vitis vinifera* with 10K genome-wide SNPs

**DOI:** 10.1371/journal.pone.0192540

**Published:** 2018-02-08

**Authors:** Valérie Laucou, Amandine Launay, Roberto Bacilieri, Thierry Lacombe, Anne-Françoise Adam-Blondon, Aurélie Bérard, Aurélie Chauveau, Maria Teresa de Andrés, Ludger Hausmann, Javier Ibáñez, Marie-Christine Le Paslier, David Maghradze, José Miguel Martinez-Zapater, Erika Maul, Maharajah Ponnaiah, Reinhard Töpfer, Jean-Pierre Péros, Jean-Michel Boursiquot

**Affiliations:** 1 AGAP, Univ Montpellier, CIRAD, INRA, Montpellier SupAgro, Montpellier, France; 2 INRA Unité Expérimentale de Vassal, Centre de Ressources Biologiques de la Vigne, Marseillan-plage, France; 3 URGV, Univ Paris-Saclay, CNRS, INRA, Evry, France; 4 EPGV, Univ Paris-Saclay, CEA, IG-CNG, INRA, Evry, France; 5 IMIDRA, Finca El Encín, Alcalá de Henares, Madrid, Spain; 6 JKI, Institute for Grapevine Breeding Geilweilerhof, Siebeldingen, Germany; 7 ICVV, CSIC, Universidad de La Rioja, Gobierno de la Rioja, Logroño, Spain; 8 National Wine Agency of Georgia, Tbilisi, Georgia; 9 LBD, Univ UPMC, CNRS, INSERM, Paris, France; National Cheng Kung University, TAIWAN

## Abstract

Grapevine is a very important crop species that is mainly cultivated worldwide for fruits, wine and juice. Identification of the genetic bases of performance traits through association mapping studies requires a precise knowledge of the available diversity and how this diversity is structured and varies across the whole genome. An 18k SNP genotyping array was evaluated on a panel of *Vitis vinifera* cultivars and we obtained a data set with no missing values for a total of 10207 SNPs and 783 different genotypes. The average inter-SNP spacing was ~47 kbp, the mean minor allele frequency (MAF) was 0.23 and the genetic diversity in the sample was high (He = 0.32). Fourteen SNPs, chosen from those with the highest MAF values, were sufficient to identify each genotype in the sample. Parentage analysis revealed 118 full parentages and 490 parent-offspring duos, thus confirming the close pedigree relationships within the cultivated grapevine. Structure analyses also confirmed the main divisions due to an eastern-western gradient and human usage (table vs. wine). Using a multivariate approach, we refined the structure and identified a total of eight clusters. Both the genetic diversity (He, 0.26–0.32) and linkage disequilibrium (LD, 28.8–58.2 kbp) varied between clusters. Despite the short span LD, we also identified some non-recombining haplotype blocks that may complicate association mapping. Finally, we performed a genome-wide association study that confirmed previous works and also identified new regions for important performance traits such as acidity. Taken together, all the results contribute to a better knowledge of the genetics of the cultivated grapevine.

## Introduction

Grape is nowadays a crop of major economic importance in the world: in 2015, around 7.5 Mha were under cultivation and produced 75.7 Mt of fruits, 56% being processed for wine, juice and spirits and 44% used for fresh or dried raisin consumption [1]. Historical and archaeological records indicate that the cultivated grapevine, *Vitis vinifera* subsp. *vinifera*, was domesticated from the dioecious taxon *V*. *vinifera* subsp. *sylvestris* [2–4], the two subspecies now diverging for several traits [5]. The combined action of spontaneous hybridization, somatic variation, selection and propagation through cuttings or seeds by humans has shaped the grapevine cultivated compartment or cultivated genetic pool, which, in contrast to other domesticated species, is now more diverse and heterozygous than its wild relative [6, 7]. The early stages of cultivation with some mix of wild and domesticated forms as well as hybrids between them [8, 9] gave rise to gene flows between the cultivated and wild subspecies [10] resulting in a local contribution of wild populations to the overall structure of the cultivated compartment [3, 11, 12]. Moreover, the wide use of the most interesting parents during domestication and early selection by humans likely favored the emergence of groups of related cultivars as observed in most traditional viticultural regions [12–17].

Assessing the genetic diversity and structure of cultivated grape has been very useful to reconstruct the domestication history of grapevine [12, 18] and to maximize diversity in core-collections [19, 20]. Core-collections could then be used as either single nucleotide polymorphism (SNP) discovery panels or resequencing panels. Such knowledge is also of prime importance to identify suitable parents or samples and to analyze the genetic bases of agronomic traits through linkage mapping or genome-wide association [21, 22]. A first comprehensive attempt to classify cultivars was achieved by Negrul [23] who took into account mostly geographical information and morphological traits. He suggested that the three cultivated “*proles*” (*occidentalis*, *pontica and orientalis*) derived from two pools of *V*. *vinifera* subsp. *sylvestris* (*typica* and *aberrans*). In the last decades, the development of molecular markers allowed characterizing both the genetic diversity and structure in large samples of *V*. *vinifera* cultivars [6, 12, 18, 20, 24]. All these data are in agreement with Negrul’s morphological classification [23] and allowed us to define a large panel for association mapping [25]. Most of these studies were performed using nuclear microsatellites (nSSRs), although some explored the potential of SNP arrays [12, 20].

The origin of specific *V*. *vinifera* cultivars has also been widely investigated since the 1990’s through parentage studies using nSSRs [13, 16]. More recently, SNPs have also been used with [17] or without [12] nSSRs markers to further support the findings. The number of SNPs used varied from approx. 240 [15, 26, 27] to approx. 12000 [28–30]. Results allowed revealing or validating full-parentages (less than 10 [30] to more than 23 [14]). For kinship analysis, the main advantage of SNPs instead of nSSRs markers was the higher number of available markers, leading to higher LOD scores [15, 31] and the possibility to distinguish full-sibling *vs*. second-degree relationships [30].

A *Vitis* genotyping array containing 18K SNPs was developed during the European project GrapeReseq (https://urgi.versailles.inra.fr/Projects/Achieved-projects/GrapeReSeq) to enable diversity, structure and genome wide association (GWA) studies in grapevine [32]. This array has already been used to study the genetic relationships between cultivated and wild Georgian vines [28] and the genetic diversity and parentage in Italian cultivars [29, 30]. At a wider scale, this tool was also used to genotype 945 accessions of *V*. *vinifera* subsp. *vinifera* (syn. *sativa*) selected in four repositories of three GrapeReseq Consortium partners (France, Germany and Spain).

In the present study, we used this large genotyping data i) to determine a minimal set of SNP markers useful for cultivar identification ii) to precise their parentage relationships iii) to refine our understanding of the genetic diversity structure of the cultivated compartment iv) to obtain an overall estimate of the linkage disequilibrium decay at chromosome level and v) to perform tests of association mapping for performance traits previously measured for this sample.

## Results

A total of 945 accessions of *Vitis vinifera* were analyzed with 18K SNPs. They represented 800 distinct genotypes, among which 17 with over 50% missing data. The analyses were thus performed on 783 unique genotypes (S1 Table).

### Properties of the SNP array and cultivar identification

**Fig 1:**
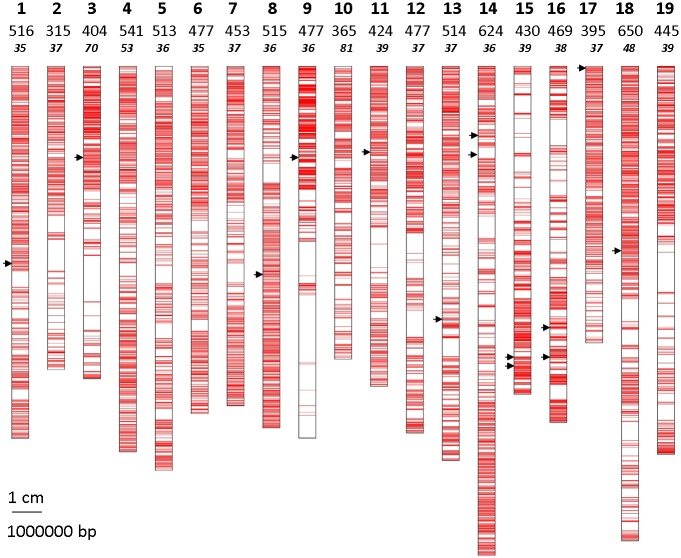
Mapping of the 9004 SNPs on the whole PN40024 reference genome sequence (assembly version 12X.V0). The 14 SNPs that allow identifying the 783 *V*. *vinifera* cultivars of this study are indicated with a black arrow. Line 1 corresponds to the chromosome number, line 2 to the total number of SNPs and line 3 to the average distance between 2 SNPs on each chromosome (in kb).

From the initial set of 18K SNPs, 14098 markers amplified and 10207 were finally retained. SNPs were rejected due to multiple clusters (73.5%), lack of polymorphism (23%) and weak amplification (3.5%). Twenty-three percent of the SNPs were mapped in exons and among them, 36% were non-synonymous. The SNP map (Fig 1) revealed some uncovered regions on chromosomes 2, 3, 9, 15 and 19, associated to a higher frequency of repeated and mobile elements [33]. On the reference genome [33], 88.2% (9004) of the SNPs mapped on chromosomes (Fig 1), 8.3% located on unmapped scaffolds (“chrUn” tag) and 3.5% on scaffolds assigned to a chromosome but not mapped (chrX_random tag). The average distance between SNPs with a minor allele frequency (MAF) >5% was around 47 kbp (min = 51 bp, max = 5915 kbp). Moreover, 1919 SNPs presented a MAF<0.1, 630 SNPs a MAF<0.05 and 102 SNPs had a MAF<0.01. On the other hand, 1508 SNPs presented a MAF>0.4. MAF distribution was balanced with a mean of 0.23 and in average heterozygosity (expected and observed) was 0.32.

The cumulated probabilities of identity (PI) calculated with FAMOZ indicated 14 SNPs as a minimum number to distinguish all 783 genotypes (Table 1). These markers were distributed along 11 chromosomes, three of which had 2 SNPs (Fig 1). MAF was equal or close to 0.5 in this set of 14 SNPs. This minimal number of markers was confirmed when data were analyzed using AMaCAID R program. Moreover, in 13 families of 2 to 5 siblings corresponding to 33 accessions, the average number of different alleles between full-sibs was 2900 (SD: 392).

**Table 1:** List and descriptive statistics of the 14 SNPs sufficient to distinguish the 783 genotypes. Positions are denoted according to the PN40024 reference genome sequence (assembly version 12X.V0). Observed heterozygosity (H_**o**_) and inbreeding coefficient (F) were calculated using PLINK software v1.9. Expected heterozygosity (H_**e**_) was 0.5 for each marker.

Chromosome	Position	Allele	MAF	H_o_	F
1	12168156	T/C	0.499	0.475	0.05
3	5724485	T/C	0.500	0.489	0.022
8	12906774	T/C	0.499	0.541	-0.082
9	5755442	T/C	0.499	0.561	-0.122
11	5406647	A/C	0.500	0.508	-0.016
13	15640723	T/C	0.499	0.480	0.04
14	4353470	A/G	0.499	0.466	0.068
14	4947068	T/G	0.499	0.447	0.106
15	18127737	T/G	0.499	0.474	0.052
15	18567587	T/G	0.498	0.512	-0.024
16	16198599	A/G	0.500	0.507	-0.014
16	17950801	A/G	0.499	0.495	0.01
17	126505	A/G	0.499	0.471	0.058
18	11544918	T/C	0.499	0.508	-0.016

### Parentage analysis

#### Parents-offspring trios

Parentage analysis on 783 cultivars using 10207 SNPs enabled validating 118 full parentages (S2 Table). LOD scores varied from 2721 to 5315 (mean 3509) and number of mismatches ranged from 0 to 9 (mean 2.8) (Table 2). Among these parents-offspring trios, 10 new relationships were detected whereas 108 full parentages had been previously published (S2 Table). Only four putative full parentages detected with nSSRs, (LOD scores 18.83 to 40.13) were either not confirmed (e.g. ‘Coarna alba’, ‘Gorgollasa’ and ‘Kadarka’) or poorly supported (e.g. ‘Gros Cabernet’) with SNPs (S3 Table).

**Table 2:** Full parentages and half kinships revealed with 10207 SNPs on 783 *V*. *vinifera* cultivars and with 20 SSRs on 701 cultivars.

	Number of relationships	LOD scores min—max	loci contribution Nb min—max	loci mismatch Nb min—max
Full parentages vs. SNP on 783 cv.	118	2721–5315	10182–10207	0–9
Full parentages vs. SSR on 701 cv.	104	18.07–55.73	15–20	0–2
Half kinkships vs. SNP on 783 cv.	490	543–2975	9512–10207	0–9
Half kinkships vs. SSR on 701 cv.	482	0.69–33.42	11–20	0–1

#### Parent-offspring duos

The analysis also validated 490 parent-offspring duos (S4 Table) among which 234 corresponded to previous trios and 256 to original relationships. LOD scores ranged from 543 to 2975 (mean 1369) and the number of mismatch from 0 to 9 (mean 1.3) (Table 2). Among these duos, 391 had been previously detected using mostly nSSRs data (S4 Table). In addition, 92 putative half kinships, previously found using nSSRs were not confirmed with SNPs having LOD scores from 0.71 to 19.75 (S3 Table).

While FAMOZ uses exclusion probabilities for parent-offspring detection, KING-robust uses a combination of identity-by-state and kinship information. We thus compared the lists of parent-offspring relationships obtained with FAMOZ and KING-robust. KING-robust validated all the parent-offspring (PO) trios and duos detected by FAMOZ.

#### Full-sibs

We also tested whether KING-robust was able to correctly identify putative full-sib (FS) pairs. Full-sib pairs were called by KING-robust when IBS0 was comprised between 0.002 and 0.25 and K was within the class range for first degree. Under these assumptions, KING-robust identified 805 putative full-sib pairs, involving 419 cultivars, 313 of which were already listed as POs. We checked this list of proposed FS using both the above results for PO duos and previous SSRs data [16]. We found that not all the proposed pairs were FS. As illustrated in the case of Gamay, among the seven proposed FS pairs, two were true FS (Gamay-Chardonnay and Gamay-Roublot), while four had only one parent in common according to the PO table and were thus half-sibs (HS: Gamay-Affenthaler, Gamay-Elbling, Gamay-Gelbhölzer Blau, Gamay-Riesling). One case (Gamay-Müller-Thurgau) was actually a special case of 2^nd^ degree relationship, because Müller-Thurgau is a progeny of Riesling and Madeleine Royale, with both Gouais blanc and Pinot noir (also the parents of Gamay) as grand-parents. Because of these discrepancies, we decided not to publish the FS list proposed by KING-robust.

#### 2nd and 3rd degree of family relationships and unrelated genotypes

Given the mixed results for the FS category, the KING-robust results for 2^nd^ and 3^rd^ degree relatives were not considered as separate categories, but rather as indicators of generic lower degree relationships to create a conservative list of “unrelated” genotypes. The lists of unrelated cultivars at 1^st^, 2^nd^ and 3^rd^ degree, including 259, 78 and 45 cultivars respectively, are presented in S5 Table. Part of this list contained cultivars having a known kinship not detected with KING-robust.

### Genetic structure analyses

**Fig 2:**
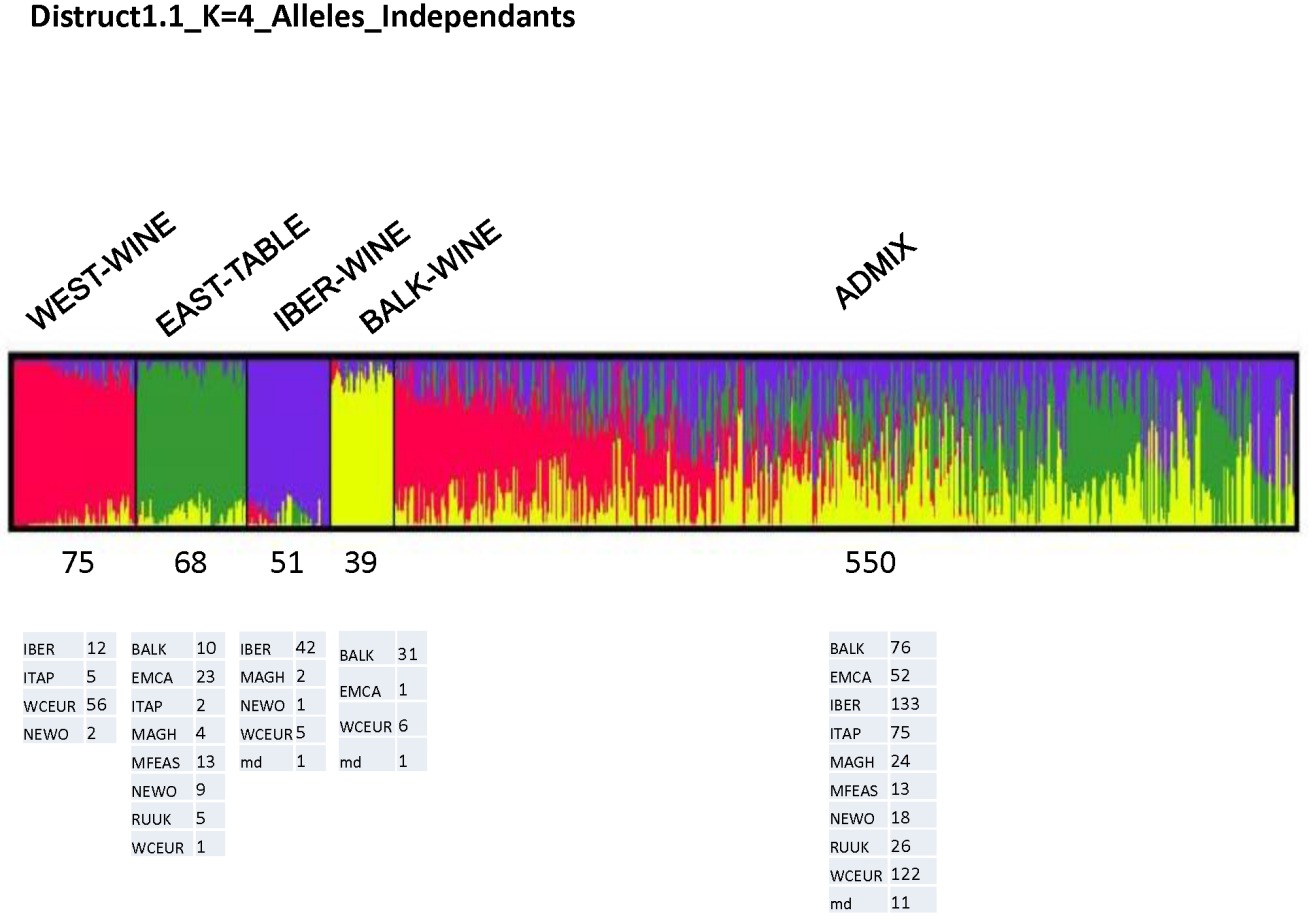
Distruct plot of Bayesian population assignments using STRUCTURE and an admixture model with independent alleles frequencies (K = 4). Each K cluster is defined by a color: K1 in pink, K2 in green, K3 in blue and K4 in yellow and number individuals and origin are specified.

Preliminary STRUCTURE runs showed that the best model for partitioning the genetic structure of the sample was the admixed model with non-correlated allele frequencies and no prior geographic information. The STRUCTURE analysis, and in particular the similarity among runs, indicated that the best subdivision of the whole dataset (783 genotypes) was obtained at K = 4 (S1 Fig). Using a threshold of >80% for cultivar assignation to subgroups, only 30% of the cultivars were included in one subgroup, and the remaining 70% were assigned to the admixed group (S6 Table). The non-admixed subgroups could be characterized as: wine grapes from the West (n = 75), table grapes from the East (n = 68), wine-table grapes from the Iberian Peninsula (n = 51), and wine grapes from the Balkan region (n = 39) (Fig 2). The Evanno delta-K statistics (S2 Fig) indicated that other subdivisions (K = 3 and K = 8) could be pertinent. The K = 3 subdivision was similar to the one already published by Bacilieri et al. [18] based on nSSR markers, so we did not report it here. For K = 8, the use of a 80% threshold for assigning genotypes to subgroups with STRUCTURE led to subgroups containing only 2 to 26 individuals, thus making their characterization difficult (S6 Table).

For the K-means clustering step in the Discriminant Analysis of Principal Components (DAPC), we retained 800 principal components (PCs) that allowed recovering 100% of the cumulated variance. The Bayesian Information Criterion (BIC) score sharply decreased to K = 4, more slowly to K = 8 and then increased beyond this value (S3 Fig). We thus retained eight prior clusters to describe the structure in our sample. The results of cross-validation obtained with the “optim.a.score” R function provided congruent indication that the optimal number of PCs was 110 for the subsequent discriminant analysis (DA) and all discriminant functions were retained (n = 7 (K-1)). The DA step conserved 58.6% of the total variance. The posterior assignment proportion varied among clusters between 95.2 to 98.8% indicating that the genetic groups identified were well separated.

**Fig 3:**
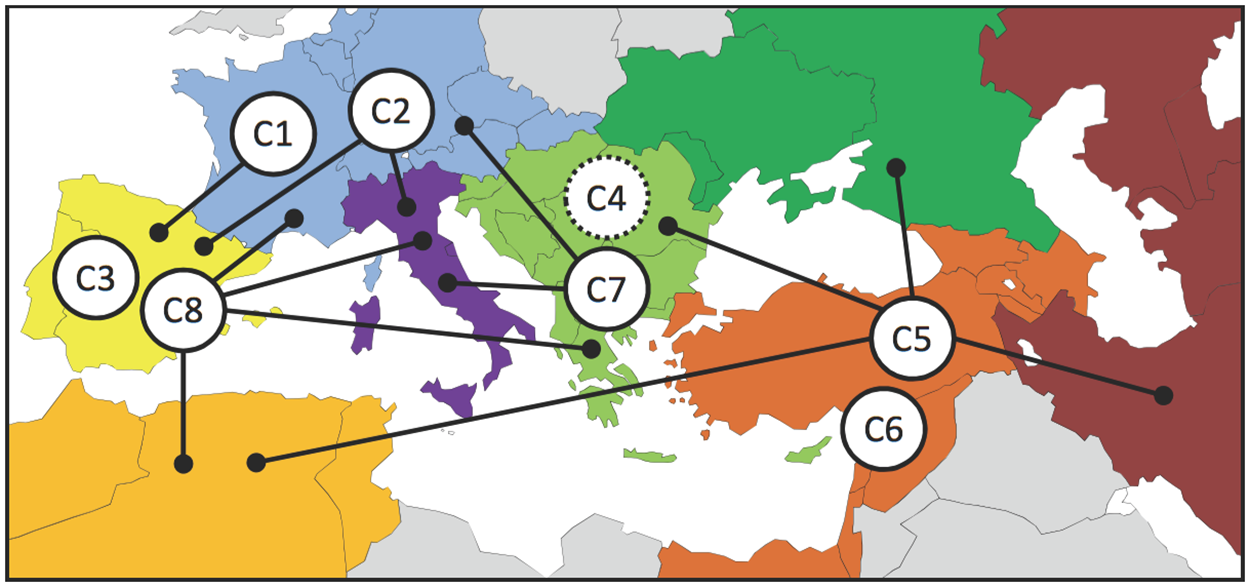
Geographical distribution of the DAPC clusters. Clusters were positioned according to the main geographic origin represented in each cluster, the contribution of other geographic zones are indicated by lines and dots (see Table 3b); seven geographic regions were considered with IBER (Iberian peninsula) in light yellow, WCEUR (Western and Central Europe) in blue, BALK (Balkans) in light green, ITAP (Italian peninsula) in purple, EMCA (Eastern Mediterranean and Caucasus) and MFEAS (Middle and Far East) in brown, RUUK (Russia and Ukraine) in green and MAGH (Maghreb) in dark yellow, cluster C4 receives a dotted line and is placed in the middle since it is essentially composed of table cultivars used mainly in the New World (NEWO), but also in several European zones (BALK, ITAP, WCEUR and IBER).

Clusters varied in size from 20 to 141 genotypes (Table 3a) and differed according to the geographical origin of cultivars (Table 3b, Fig 3) and grape usage (Table 3c):

- C1 mainly included wine cultivars from Western and Central Europe (WCEUR) and the Iberian Peninsula (IBER).
- C2 was also composed of wine cultivars from Western and Central Europe (WCEUR) and Iberian Peninsula regions (IBER) but with a notable proportion of wine cultivars from the Italian Peninsula (ITAP).
- C3 included predominantly wine cultivars and few table grape from the Iberian Peninsula.
- C4 had a high percentage of table cultivars from diverse Western European regions and contained most of the new breeding table cultivars assigned to the New World group (NEWO).
- C5 contained cultivars from very diverse regions but mostly table cultivars from Eastern regions.
- C6 included a low number of wine cultivars from the Eastern Mediterranean and Caucasus regions (and especially from Georgia).
- C7 was centered on the Balkan region but also included cultivars from Western and Eastern regions, most of them being wine grape cultivars.
- C8 included a large proportion of Iberian cultivars but also cultivars from WCEUR, BALK and ITAP regions and around one third of all cultivars from Maghreb.

**Table 3:** Description of the identified DAPC clusters.

DAPC cluster	C1	C2	C3	C4	C5	C6	C7	C8	
**a. Size, genetic diversity**^a^									**Global**
No. Genotypes	80	141	98	52	135	20	125	132	783
He	0.304^C^	0.321^A^	0.288^E^	0.282^E^	0.285^E^	0.276^E^	0.300^D^	0.309^B^	0.32
Fis	-0.108	-0.054	-0.125	-0.051	-0.017	-0.084	-0.051	-0.05	-0.06
**b. Genotype distribution according to their geographic origin**^b^									**Total (%)**
IBER	20	22	88	4	4	0	1	63	202 (25.7)
WCEUR	53	74	5	6	2	0	13	24	177 (22.8)
BALK	2	2	0	10	17	1	70	10	112 (14.4)
ITAP	1	35	0	7	3	0	20	16	82 (10.5)
EMCA	0	1	0	1	48	17	8	2	77 (9.8)
MFEAS	0	0	0	0	26	0	0	0	26 (3.3)
RUUK	2	1	0	0	15	1	9	2	30 (3.8)
MAGH	0	2	3	1	12	0	0	11	29 (3.7)
NEWO	2	0	1	21	4	0	0	1	29 (3.7)
nd	0	4	1	2	4	1	4	3	19 (2.4)
**c. Genotype distribution according to their human usage**^c^									**Total (%)**
Wine	71	120	72	4	25	12	89	85	478 (61.1)
Table	4	4	10	43	90	1	15	26	193 (24.6)
Double	5	13	16	5	15	0	21	21	96 (12.2)
nd	0	4	0	0	5	7	0	0	16 (2.0)
**d. Genotype distribution with structure K = 4**^d^									**Total (%)**
Wine West	20	55	0	0	0	0	0	0	75 (9.6)
Table East	0	0	0	8	60	0	0	0	68 (8,7)
Wine Table IBER	0	0	50	0	0	0	0	1	51 (6.5)
Wine BALK	0	0	0	0	0	0	39	0	39 (5.0)
Admixed	60	86	48	44	75	20	86	131	550 (70.2)

^a^Genetic diversity was calculated as the expected heterozygosity (He) in each cluster and the global sample using adegenet. Difference in He between clusters was tested with adegenet (Hs.test with n.sim = 500) and clusters having significant difference at P<0.05 were placed in different groups (capital letters). The inbreeding coefficient (Fis) was calculated using the hierfstat R package with 100 bootstraps.

^b^Geographical groups according to Bacilieri et al. (2013)[18]. IBER: Iberian peninsula, WCEUR: Western and Central Europe, BALK: Balkans, ITAP: Italian peninsula, EMCA: Eastern Mediterranean and Caucasus, MFEAS: Middle and Far East, RUUK: Russia and Ukrain, MAGH: Maghreb, NEWO: New World Vineyard, nd: not determined.

^c^Cultivars were used to produce wine, table consumption or for both usages, nd: not determined.

^d^Assignment based on the STRUCTURE analysis performed in this study

The DAPC grouping was highly consistent with the results obtained using STRUCTURE for the 233 non-admixed genotypes (Table 3d). The genotypes assigned by STRUCTURE to the Wine West group, Table East group, Wine Table Iberian group and Wine Balkan group, were included in C1 or C2 cluster, in C4 or C5 cluster, in C3 cluster, and in C7 cluster, respectively. In contrast to STRUCTURE, DAPC allowed assigning all genotypes and permitted a clear identification of both the eastern cluster C6 (mostly Georgian cultivars) and the western cluster C8 (mostly Iberian and Maghreb cultivars).

**Fig 4:**
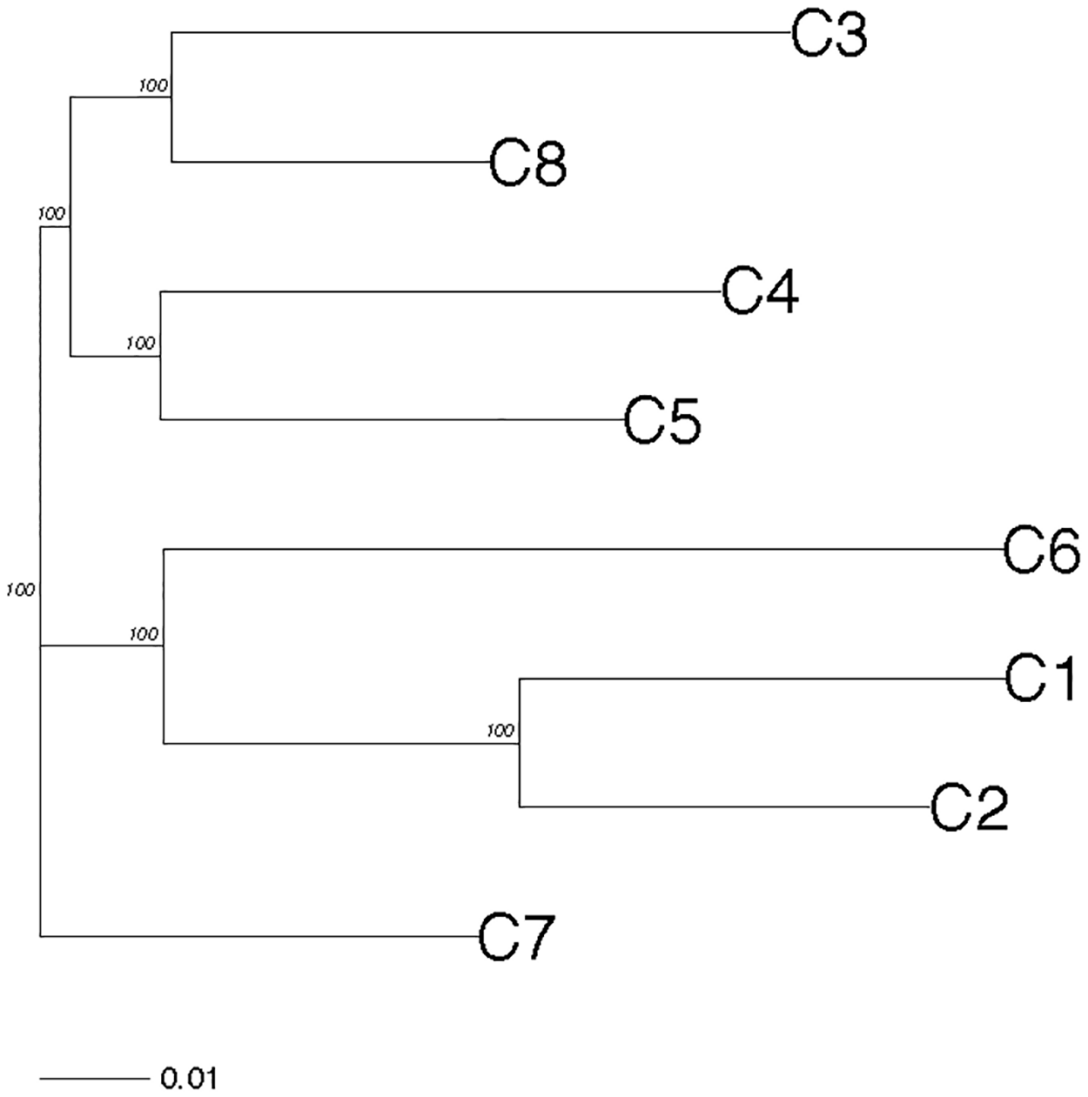
Dendrogram based on the Roger’s distance between DAPC clusters. All nodes received a 100% bootstrap support.

Visual inspection of the scatterplots of the DAPC analysis showed that some clusters such as C3, C6 and C4 were more easily distinguishable than others (S4 Fig). The different measures (Gst, G’st and D) used to evaluate the genetic differentiation between clusters were highly correlated (pairwise Kendall’s tests) and we only present the results with the most used parameter, Nei’s measure. Pairwise Gst varied from 0.016 to 0.064 (Table 4) and G tests indicated that all values were highly significantly different from zero (P<0.001). Using Roger’s distance, all the clusters were also clearly distinguished with a maximal bootstrap value at each node (Fig 4).

**Table 4:** Pairwise Nei’s Gst^a^ between DAPC clusters.

**DAPC cluster**	**C1**	**C2**	**C3**	**C4**	**C5**	**C6**	**C7**
**C2**	0.016						
**C3**	0.059	0.051					
**C4**	0.055	0.05	0.041				
**C5**	0.061	0.055	0.039	0.024			
**C6**	0.06	0.047	0.064	0.06	0.042		
**C7**	0.039	0.035	0.033	0.029	0.023	0.04	
**C8**	0.039	0.03	0.018	0.024	0.02	0.042	0.016

^a^ all values were highly significant (P<0.001) based on exact tests for genotypic differentiation.

We performed additional analyses to evaluate the robustness of the clustering obtained with DAPC. First, in order to reduce the first degree of kinship in the sample, the offspring in each trio was discarded, resulting in a new file with 665 genotypes. The decrease in size was different for the eight clusters, evidencing different levels of kinship within each one of them. Cluster C6 retained the same size (no related individuals), some clusters were only weakly affected (size reduction of 9.9%, 10.3%, 8.9% and 8.5%, for C2, C5, C7 and C8, respectively), while others were more severely affected (size reduction of 28.4%, 26.6% and 36.5% for C1, C3 and C4, respectively). K-means clustering with the same settings than above identified seven clusters as the most probable subdivision. Prior groups from the two analyses were compared in a contingency table. Both arrangements were mostly congruent, with the majority of genotypes in the eight clusters still being grouped together in one of the seven clusters (S5a Fig). Second, we combined the data of the 783 samples with those obtained by De Lorenzis et al. [28] using the same SNP genotyping array. After filtering against missing data, a new data set was constructed that included the initial 783 genotypes and 41 Georgian cultivars, all being genotyped with 8096 SNPs. K-means clustering again identified eight clusters. The 783 initial genotypes were grouped in the same previous clusters whereas most of the additional (35) Georgian cultivars were placed in cluster C6 (S5b Fig).

### Genetic diversity

Within-cluster genetic diversity (measured as expected heterozygosity He, Table 3a) varied from 0.276 to 0.321, with 0.320 for the global sample of 783 genotypes. The pairwise differences in He between clusters presented significant differences with C2, C8, C1, C7 clusters showing different and high values, whereas C3, C5, C4, C6 had close and lower values. The departure from panmixia at the subpopulation level (Fis, Table 3a) was larger in C3 (composed of a majority of Iberian Peninsula cultivars), with an excess of heterozygous SNPs, while C5 (with a majority of Caucasian cultivars) was close to equilibrium. The Fis coefficient pointed to a consistent overall heterozygous excess both at the subpopulation and at the whole population level (-0.06). At the individual level, a higher heterozygosity (Individual He, S6 Table) was observed in traditional wine cultivars from the West (e.g. Chouchillon, Verduzzo Friulano, Carrasquin, etc.), while higher inbreeding was present in some Eastern table cultivars as well as in recent table grape crossbreds such as Breider 5–6 (self-pollination), Red Globe, Flame seedless, Fiesta or Victoria, all of which have complex pedigrees involving inbreeding. Individual heterozygoty (Fis) largely varied from -0.218 to 0.466 (S6 Table).

### Linkage disequilibrium analysis

**Fig 5:**
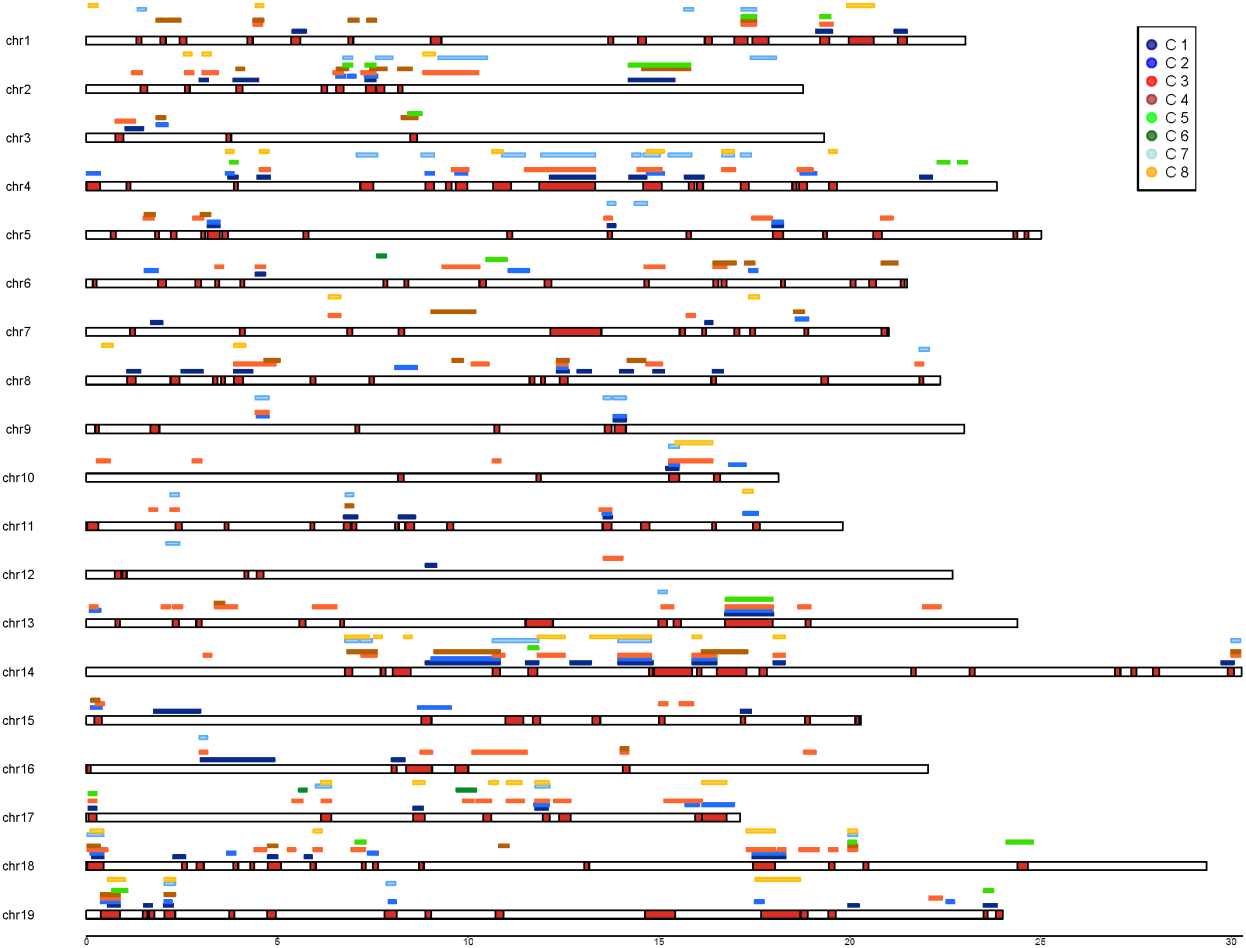
Haplotype non-recombinant blocks along chromosomes. Only blocks including more than 5 SNPs and larger than 100 kbp in length are represented. Red regions along the main chromosome lines denote non-recombinant blocks calculated on the whole panel of 783 genotypes. Colored bars above it represent non-recombinant blocks calculated on each of the 8 DAPC subgroups. Chromosome length on X axis is in Mb.

Linkage disequilibrium (LD) was evaluated in each DAPC subgroups. LD range varied between 28.8 kbp and 58.2 kbp according to subgroup (S6 Fig). The longest LD was found for the C6 subgroup, which is the one with the lowest genetic diversity but also the smallest subgroup (n = 20). Non recombining haplotype blocks were found spanning as much as 17 SNPs and 1,950 kbp in these subgroups. These blocks were covered by a SNP density similar to the SNP density over all chromosomes (on average, one SNP every 41 kbp in these blocks compared to 47 kbp for the whole genome), and were not correlated to putative centromeric regions (A. Canaguier, personal communication). Some blocks were consistently found across subgroups, sometime characterizing large regions (e.g., on chromosome 14 Fig 5).

### Application to genome-wide association studies (GWAS)

Genome-wide association analysis (GWA) using the MLM model, corrected for both structure and kinship, provided interesting association results for some performance traits at the standard Bonferroni p level (Table 5). We detected signals for budburst-to-veraison time, cluster weight, Muscat flavor, wine acidity, seedlessness, berry skin color, and sex. The signal for some traits was found only in one or few SNPs on a single chromosome (ex. only one SNP associated with Muscat flavor on chromosome 5), while other traits displayed a more complex disposition (such as seedlessness, with associated SNPs on three different chromosomes, or berry skin color, with forty associated SNPs spanning a 10 Mbp region on chromosome 2).

**Table 5:** Summary of the signal detected by GWAS. In the "Note" column, we indicate the number of associated SNPs if any. In the "Literature" column, we report existing publications consensual to our findings. Berry skin color, Muscat flavor, seedlessness and sex of flower were scored as qualitative traits.

Trait (OIV descriptor)	Significance at Bonferroni Threshold p < 5.00E-06	Probability level at which a signal is detected	Chr n.	Note	Literature
Berry weight (OIV-503)	No				
Time bud burst (OIV-301)	No				
Time bud burst to full bloom	No				
Time bud burst to ripeness	No				
Fertility (OIV-155)	No				
Must acidity (OIV-506)	No				
Susceptibility to *Botrytis* (OIV-459)	No				
Utilization of fruit	No				
Wine % of alcohol	No				
Budburst to veraison time	Yes	1.00E-06	3	1 SNP	
Cluster weight (OIV-502)	Yes	2.00E-07	13	1 SNP	
Muscat flavor (OIV-236)	Yes	1.00E-09	5	1 SNPs near the VvDXS gene	Emanuelli et al. 2010, Doligez et al. 2006, Duchêne et al. 2009, Battilana et al. 2009 (chr5)
Wine acidity	Yes	5.00E-11	2	7 SNPs between 12.5 and 16.8 Mbp	
Seedlessness (OIV-241)	Yes	5.00E-15	6, 9, 19	8 SNPs in chr6 between 9.5 and 12.2 Mbp	Costantini et al. 2008 (chr6)
Berry skin color (OIV-225)	Yes	3.00E-77	2	40 SNPs between 9 and 19 Mbp	Fournier-Level et al. 2009, Huang et al. 2013 (chr2)
Sex of flower (OIV-151)	Yes	9.00E-89	2	6 SNP across the sex locus	Picq et al. 2014, Fechter et al. 2012 (chr2)

**Fig 6:**
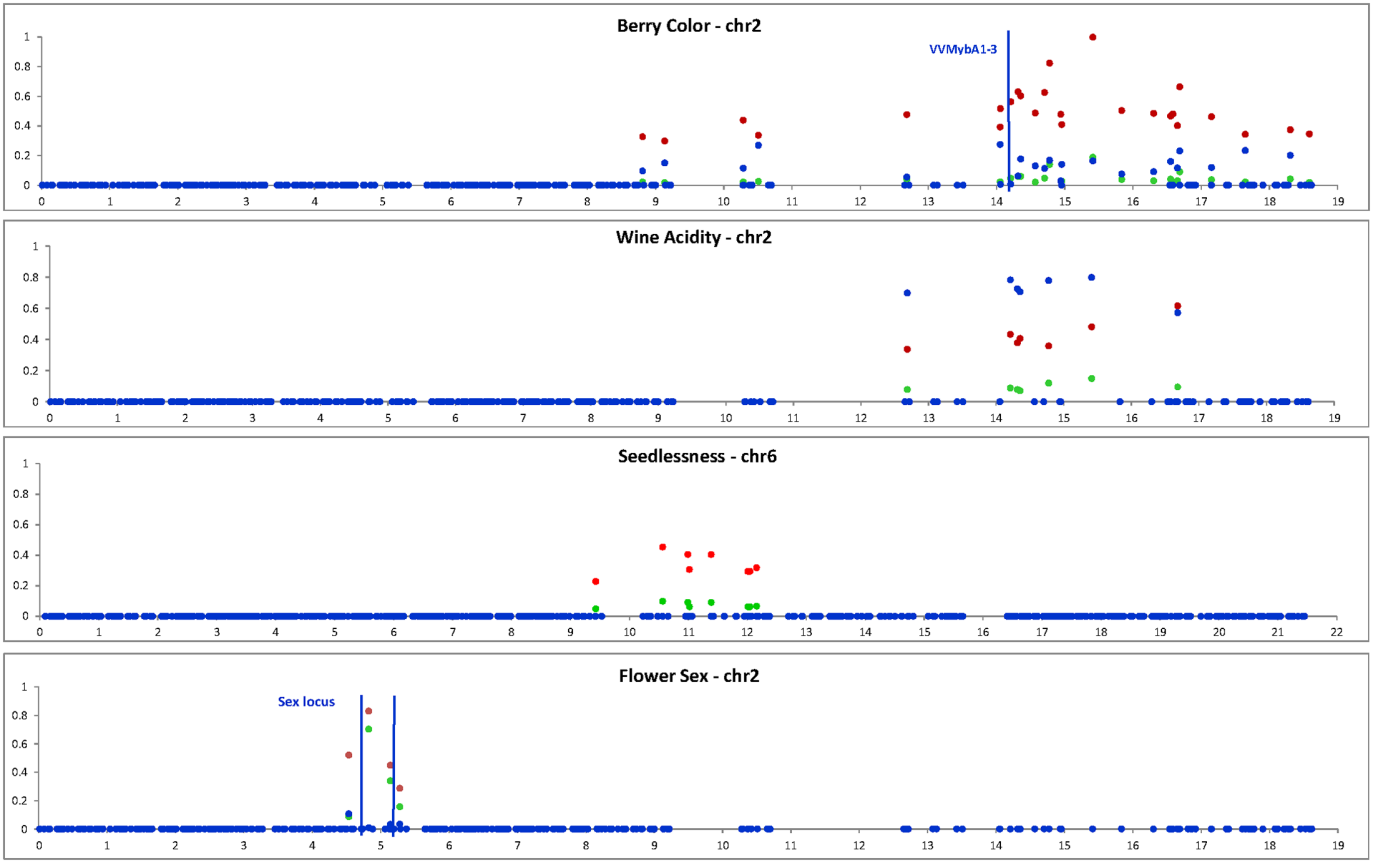
Association signals for performance traits detected in the studied panel. Green dots are the r2 values, while red and blue dots represent the effect of the major and minor allele, respectively. The location of a gene cluster associated with berry skin color (VVMybA1-VVMYbA3, Fournier-Level et al. 2009 [34]) and the boundaries of the sex locus (Fechter et al. 2012 [35], Picq et al. 2014 [36]) are indicated.

In Fig 6, we provide a graphical representation of the associations involving multiple SNPs on a chromosome.

## Discussion

The availability of a new molecular tool, the *Vitis* 18K genotyping array, allowed us to explore several characteristics in a large sample of grape genetic resources. The accessions studied in this work originated from four major European repositories and represent a large number of cultivars that have already been studied using either morphological traits or microsatellite markers [24, 26, 37, 38]. The availability of the 18K SNP array allowed a more exhaustive exploration of this precious genetic pool with between 315 and 650 markers per chromosome.

### Minimal set of SNP markers useful for cultivar identification

In grapevine, a 9-SSR genotyping system has been established as a reference tool, to identify thousands of cultivars in Europe and neighboring countries [38]. However, SSR genotyping is subject to technical variations that necessitate precise calibration between laboratories [39]. An SNP array was first proposed as an alternative for cultivar identification by Cabezas et al. [40]. These authors used a set of 48 SNP markers with a low probability of identity (PI) value (1.4 10^−17^) to discriminate 200 cultivars. In the present work, we found that 14 SNPs only are sufficient to distinguish the 783 genotypes representing most of the cultivated compartment diversity. The probability of identity obtained here was higher than that calculated with 9 SSR markers (7 10^−6^ vs. 4 10^−11^, [24]). In addition, Lijavetzky et al. [41] estimated that 20 SNPs were needed to reach a PI (4.10^−9^) similar to that obtained with the six SSRs included in the OIV reference set. The size and composition of minimal SNP sets depend both on the genetic diversity and the relatedness in the genotyped sample. However, using the same 18K SNP array, a minimal set of 12 SNPs has been found by Mercati et al. [29] to distinguish a less diverse genetic group of 101 Sicilian grapevines. To reach the same level of theoretical number of possible profiles as the one obtained with the set of 9 SSRs, around 40 SNPs would be necessary. These 40 SNPs should have a MAF close to 0.5 and be well distributed on the 19 grape chromosomes; they could be selected among the 740 SNPs of this panel having a MAF > 0.45 or among the 326 SNPs having a MAF > 0.48.

Although microsatellite markers are most adapted for cultivar identification (high PIC, fast results, easy to assay, cost effective, available databases), several research groups are currently using the 48-SNP set previously defined for that purpose [20, 40, 42]. There are indeed hundreds of accessions already genotyped with these SNPs [14, 17, 27, 31]. Twenty-six SNPs were found in common with the 48-SNP set. Given that the technologies to genotype large numbers of DNA samples with few SNPs are already available [43, 44], SNPs will become the markers of choice for grapevine cultivar identification once it is possible to analyze at a reasonable cost small numbers of samples, as it is often the case for identification purposes. However, for SNPs to be used in routine identification, large databases similar to those already available for SSRs (http://www.vivc.de/index.php) are needed.

### Parentages

Several confounding factors may complicate parentage studies in grape: (i) genetic structure, (ii) generation overlapping due to vegetative propagation and use of the same cultivar in several breeding generations [16], (iii) sharing of genitors across genetic pools and (iv) possible confusion between identity by descent (IBD) and identity by state (IBS) for traditional cultivars having no breeder’s records. Concerning genetic structure, we observed that removing all cultivars (118) having both their parents in the initial set of 783 cultivars did not modify the observed genetic structure. Concerning the last point, given the high number of markers used, the low number of generation generally admitted for grapevine [12, 16] and the confirmation of already known pedigrees, we argued that parentages established here correspond to IBD.

Most of the 118 parents-offspring trios had already been detected in previous studies mainly based on SSRs markers, but it was achieved here with higher LOD scores. The ten new trios detected (mostly for Spanish cultivars) corresponded to accessions not included in previous parentage studies (e.g. Breval negro). Finally, 10207 SNPs invalidated only 5% of the trios based on 20 nSSRs. We therefore argue that using around 30 nSSR loci, as recommended by Cipriani et al. [45] would probably have led to the same discoveries than using 10207 SNPs.

The situation is different for parent-offspring duos (PO) with 19% of putative half-kinships based on 20 nSSR data finally invalidated with 10207 SNPs. Thus, our results confirmed other studies [30] on the utility of large SNPs datasets to validate close relationships and distinguish PO from other degrees of relationships.

These other degrees of relationship could not be addressed using FAMOZ and we used KING-robust that however provided mixed results for full-sibs (FS), 2^nd^ and 3^rd^ degree relationship categories. While the kinship estimations (K values) can be trusted, the exact family relationship was shown to be sometimes roughly estimated when the pair had two common ancestors instead of being FS. The kinship coefficient distribution allowed us to identify new unrelated cultivars (S5 Table). The absence of relationship could be explained by the absence of related cultivars in the sample studied (e.g. Courbu) or because recent crossbreds such as Crimson seedless or Fiesta created from unrelated parents have so far never been used as genitors [16]. A part of these unrelated cultivars can also be seen as resources that have been only rarely (or never) included in breeding, either because they are rare and abandoned, or because they did not show any favorable traits in earlier tests. In spite of this and after evaluation, they may still carry performance traits useful for pre-breeding [16].

These parentage results together with diversity analysis were of interest both for elucidating origin and relationships of grape cultivars, and for GWAS.

### Population structure

Population structure was detected within the cultivated compartment of grapevine as in previous studies [6, 18, 20]. This structure was mostly impacted by two factors that are difficult to extricate from each other: geography with an eastern-western gradient and human usage with wine or table as already identified by Negrul [23] using morphological traits. Human dissemination of cultivars, selection of descendants from few genitors and vegetative propagation resulted in a rather homogeneous pool of cultivated genotypes with little differentiation between subgroups. In the sample studied here, STRUCTURE software was able to capture those main subdivisions according to geography and usage. However, a large number of genotypes were not assigned and remained in a large admixed group. This could be due to several factors such as: i) the low genetic differentiation [46]; ii) a departure from underlying assumptions of the Bayesian model (random mating population) which are probably not met in this cultivated grape compartment; iii) a true admixing in some cultivars if they directly descend from spontaneous or man-made crosses between parents belonging to separate ancestral groups; and finally iv) a computational difficulty for STRUCTURE to assign individuals to groups in presence of a very large number of informative markers. Furthermore, on such a large data set STRUCTURE required a considerable computational time.

We therefore completed the STRUCTURE analysis with a multivariate approach that combined K-means clustering for detecting clusters and discriminant analysis to summarize differentiation between clusters (DAPC analysis [47]). Using this method, we identified eight well-differentiated clusters that captured the previous information we had on the cultivated compartment of *V*. *vinifera*. In addition, we detected some original groups and increased resolution for others. First, a group (C6) containing mostly Georgian cultivars has been identified in Eastern Europe. Adding cultivars genotyped using the same SNP array [28] allowed us to confirm this cluster using a larger number of Georgian cultivars. This also demonstrated that such an SNP array easily permits combining data from different studies in contrast to the important standardization work needed with nSSRs [39]. Another original result was the assignment of cultivars from the Iberian Peninsula in two different clusters. One (C3), also detected by STRUCTURE, corresponded to cultivars specifically originated from the Iberian Peninsula and a second one (C8), more diverse, to cultivars from Iberian Peninsula but also from other regions including the Maghreb area. These groups did not correspond to the two cultivated groups identified by De Andrés et al. [48] using STRUCTURE on 181 European cultivars for 20 nSSR markers. Nearly a hundred Iberian accessions were common to both studies and indeed distributed mostly in C3 and C8 but at similar frequencies (data not shown). In addition, cultivars from Western and Central Europe were also separated in two clusters (C1 and C2), the second containing cultivars from the Italian Peninsula and displaying less inbreeding. The cultivars used for table grape consumption were mostly assigned to clusters C4 and C5. The C4 cluster contained genotypes diffused worldwide and intensively used for breeding as evidenced by the high inbreeding rate observed in this cluster. Amongst the cultivars with a table usage not assigned in clusters C4 and C5, we found a small group of eight cultivars assigned to cluster C1 and C2 corresponding to the “Madeleine” parentage group described by Lacombe et al. [16]. The other table cultivars were assigned to clusters C3, C7 and C8 indicating probably several diffusion routes from the East. Finally, the rather well differentiated cluster C7 mainly contained cultivars from the Balkan region.

The domestication process of *V*. *vinifera* may partly explain the original structure identified here in a large cultivated sample. The eight DAPC clusters indicated a complex domestication history with more secondary domestication events. The genome-wide diversity study published by Myles et al. [12] confirmed a putative initial domestication in the Near East [4] and pointed out the relatively weak bottleneck experienced by grapevine during domestication, as well as the probable introgression from local wild *sylvestris*. This latter point has been also suggested by De Andres et al. [48] to explain the high ancestry values observed in their study between wild Spanish populations and cultivars. In addition, they found two differentiated groups of wild individuals that probably resulted from several Iberian refuges as already demonstrated for other taxa [49]. It is therefore possible that the structure of the *V*. *vinifera* cultivated compartment is due to separate contributions of diverse wild populations as previously suggested [11, 50]. This may explain the differentiation found both in the Iberian Peninsula (C3 and C8) and in Western and Central Europe (C1 and C2). To have a deeper insight into the structure of the cultivated compartment it seems therefore important to include in future studies wild populations encompassing their full range of distribution.

### Genetic diversity

The analysis of genetic diversity at the DAPC subgroup level showed more diversity in central grape growing regions, and less in peripheral ones. Some genotypes with a higher inbreeding rate than expected could be found among Eastern and also modern cultivars, most probably due to the use of family-related cultivars as parents.

However, the most striking result was the overall heterozygous excess found in spite of the widespread family structure within the 783 studied genotypes, which were connected respectively 67% and approx. 92% by first- and second-degree family relationships. While it could be expected that such a family structure had produced an overall heterozygote deficit (inbreeding), a heterozygote excess was observed on average both at the subpopulation and at the whole population level. This finding supports the hypothesis, recently proposed by Zhou et al. [51] of a high level of genetic load in grapevine, with a number of deleterious alleles that would remain silent in the heterozygous state, and that would be counter-selected in the homozygous condition. Before grape domestication and the spreading of the hermaphrodite mutation, ancestral wild grapes were dioecious, which is an efficient system to avoid direct inbreeding [52, 53], but which also promotes the building-up of a genetic load [54, 55].

This hypothesis could explain both the weak genetic bottleneck in grape [12] and the high diversity observed in cultivated grape as compared to its wild relative *V*. *vinifera* subsp. *sylvestris* [6, 20, 24, 50]. Inbreeding depression in grape has been reported by breeders [56–58] and very few cultivars indeed arose from self-pollination. Due to its implications for vine improvement, the genetic architecture of inbreeding depression (how many loci, which traits) has to be addressed in depth in future studies.

### Linkage disequilibrium

The linkage disequilibrium between adjacent SNPs showed a rapid decay, on average dropping to less than *r*^2^ = 0.2 at a distance varying between 29 and 58 kbp among the DAPC subgroups. This rapid decay is comparable to earlier findings in grapevine [12, 25, 44]. The LD being correlated to the number of recombination events along generations, lower or higher LD within subgroups may testify to different group histories. The three subgroups with the fastest LD decay were the Table-East (C5), the Balkan (C7) and Iberian (C3) subgroups. However, the LD calculation is sensitive to both the effective population size and the choice of the chromosomic regions [25]. In addition, in our case, the LD subgroup curves are not significantly different from one another. Thus, any conclusion of a possible casual relation among linkage disequilibrium and group history must be taken with caution.

On the other hand, we also found large variation among chromosome regions and SNP pairs, with, in some cases, LD extending over regions as long as 2 Mbp, a pattern that can be partly explained by a combination of breeding, selection and vegetative reproduction. The occasionally long non-recombining regions may prevent the genetic fine mapping of a trait by association genetics, if the genes determining the trait are located in these regions.

### Application to genome-wide association studies (GWAS)

GWA analysis provided interesting, and in one case unprecedented, association results for some performance traits. The SNPs associated with Muscat flavor co-localized with one region, and in particular with the VvDXS gene, already described by several previous publications [59–62]. The sex region was also well identified consistently with previous works [35, 36].

GWA identified a locus for seedlessness on chromosome 6, partially co-localized with one of the QTLs identified by Costantini et al. [63], but it failed to detect the major locus for seedlessness identified in former studies [64, 65] near the VMC7F2 SSR marker on chromosome 18. This can be probably due to the low number of seedlessness grape cultivars (n = 26) present in the 783 cultivars panel, most of them members of the “Sultanine” family group. Indeed, the SNP closer to VMC7F2 (chr18:26914334) displayed a genotype frequency in the seedless grapes (CC/CT/TT = 5/20/1) very different from its frequency in the rest of the panel (26/233/461). However this difference was not significant in the kinship-corrected GWAS test. The only seedless cultivar with a TT genotype was “Corinthe noir”, which is believed to harbor a genetic determinism of seedlessness unrelated to the one of the “Sultanine” group.

A large number of SNPs were associated with the berry skin color trait, spanning a surprisingly large 10 Mbp region around the VvMyb gene cluster [34]. There are several copies of Myb genes on chromosome 2 but a blast reveals that they span around 200 kbp only. With a similar approach, Myles et al. [12] also found on chromosome 2 a large region associated with color in grape (5 Mbp in their case). According to Fournier-Level et al. [66], the white allele mutational pattern carries the signature of a recent phase of strong exponential expansion, with a fast diffusion of the original allele in the modern germplasm. A strong expansion in a few generations implies a small number of recombination events. In addition, the white trait is recessive, and, within locally homozygous white cultivars, recombination may even be lowered. Thus, the long region associated with color may be due to a small number of recombination events since the first selections on color.

Association mapping revealed one SNP linked to cluster weight on chromosome 13, not coinciding with any previously described QTL for this trait. In previous works based on bi-parental crosses [67, 68], cluster architecture was described as having a complex genetic determinism involving many loci on at least 6 chromosomes. In our case, the complex genetic structure of our large panel, with probably several independent mechanisms determining cluster weight in different genetic backgrounds, may render the detection of SNP associations with cluster weight even more difficult.

Similar reasons may lay behind the lack of signal for phenology: we found only one SNP weakly associated with budburst-to-veraison time, while no association was found for other phenological traits. In this case too, previous works highlighted many different loci putatively linked to phenology, over many chromosomes, with little or no consensus among publications [63, 69–73]. A complex genetic architecture, with a high level of Genotype x Environment x Year interaction probably underlies phenology traits determination.

For the first time to our knowledge, we found on chromosome 2 an association between SNPs and wine acidity, with a quite high statistical support (*p*<5.00E-11, *r*^2^ = 0.15, 7 SNPs over 4.4 Mbp). However, our analysis failed to detect any association with must acidity, a fact probably linked to the measurement methodology of must acidity. In fact, according to must storage conditions (T°, time, amount of skins and hence potassium), the quantity of tartaric precipitation may vary greatly, impacting the measure of titratable acidity. This phenomenon is probably less critical in wine, where tartaric precipitation is expected to be homogeneously completed in the tanks.

Globally the figure derived from GWAS was that, given the current number of SNPs and samples, it is possible to find associations with monogenic traits; but when the genetic determinism is more complex, with a strong environmental interaction, a much higher number of SNPs, a specifically designed experimental layout, and a more precise methodology for measuring phenotypes are clearly needed.

In addition, the finding in this large and diverse panel of some quite long non-recombining chromosomal blocks may also hinder somehow fine-mapping efforts, should the underlying genes of interest be located in those regions. In our opinion, large intercrossing programs should be developed to provide access to new genetic variability and haplotype recombinants.

## Conclusions

The currently available large number of markers spread all over the whole genome opens new prospects for a better understanding of *V*. *vinifera* evolutionary history and diversity, and also to advance our understanding of the genetic potential of cultivars. Knowledge of the allelic diversity present in grapevine genetic resources is a real challenge for the future, in particular for all breeding effort, whether through traditional hybridization or New Breeding Technologies. SNPs are the markers of choice for gene discovery, linkage and QTL analyses and GWAS. Compared to microsatellite markers, they have the advantage, in addition to their number and distribution, to yield results that are much easier to transfer. In this respect, they can serve to improve the traceability and identification of the plant material. Nevertheless, the success of their use in this field as a substitute for microsatellite markers will depend on the means mobilized at international level to continue developing reference database(s) and the ability of the techniques to provide genetic profiles at the lowest cost. In a more general way, the success of single nucleotide uses will depend on the understanding of the genotype-phenotype correlations, especially for complex traits governed by polygenic architecture, genotype environment interactions and low heritability.

The work presented here contributes to enlarge the existing database and makes it possible to propose a large number of additional SNPs markers for genetic questions and a sound basis for the expansion of a database similar to the VIVC database for SSRs (http://www.vivc.de/index.php).

## Materials and methods

### Plant material and DNA extraction

Plant material originated from four grapevine collections (see S1 Table for their detailed contributions in term of accessions and characterization data):

- France, “INRA Domaine de Vassal, Marseillan-Plage” http://www6.montpellier.inra.fr/vassal; FAO WIEWS institute code FRA139
- Spain, “IMIDRA Finca El Encin, Madrid” http://www.madrid.org/coleccionvidencin; FAO WIEWS institute code ESP080
- Spain, “ICVV, Logroño” http://www.icvv.es; FAO WIEWS institute code ESP217
- Germany, “JKI Geilweilerhof, Siebeldingen” http://www.deutsche-genbank-reben.jki.bund.de, FAO VIEW Institute code DEU098

Total DNA was extracted by each partner with Qiagen DNeasy Plant Mini or Maxi Kit (Qiagen, Hilden, Germany), according to the manufacturer’s instructions except that 1% polyvinylpyrrolidone (PVP 40,000) and 1% β-mercapto-ethanol were added to the AP1 buffer. DNA was quantified with Quant-it Picogreen dsDNA Assay Kits (InVitrogen, Life Technologies).

### Description of the 18K SNP choice, characteristics, genome distribution

Within the GrapeReSeq project, SNP discovery was carried out using low-coverage, full sequencing of a panel of domesticated and wild *Vitis* genotypes [32]. An average of 8.1+/-2.9 Gb of 101 bp Illumina reads were obtained for 43 *V*. *vinifera* subsp *vinifera*, four *V*. *vinifera* subsp *sylvestris*, three *V*. *cinerea*, three *V*. *berlandieri*, four *V*. *aestivalis*, three *V*. *labrusca* and five *Muscadinia rotundifolia genotypes*. A pipeline termed MAPHiTS (**M**apping **A**nalysis **P**ipeline for **H**igh-**T**hroughput **S**equences, http://urgi.versailles.inra.fr/Tools/MAPHiTS) was used to map the short reads on the PN40024 reference genome and to detect polymorphisms. From over 4.3M SNPs initially detected, 18K SNPs were chosen according to several criteria: 1) no variation along 60 bp in both the 3’ and 5’ directions (Illumina specificities) 2) SNPs in regions involved in structural variations and repetitions were filtered out 3) the remaining SNPs were then selected based on their even physical repartition along the 12X.V0 version of the genome (FN597015-FN597047 [33 entries] at EMBL, release 102) together with their minor allele frequency (MAF) (http://urgi.versailles.inra.fr/Species/Vitis/GrapeReSeq_Illumina_20K).

### Genotype calling and SNP checking

Genotype data were scored and validated from the GrapeReSeq 18K *Vitis* genotyping chip raw data using GenomeStudio Data Analysis v2011.1 (Illumina Inc, San Diego, CA, USA). The sequenced reference accession PN40024 was included as genotype control for allele standardization. In this process, we discarded SNPs that were monomorphic, with low amplification, more than 3 clusters, or missing data, as well as chloroplast SNPs. We obtained a data matrix of 10207 SNPs and 783 unique individuals with no missing data among our initial sample of 945 cultivars.This dataset is available at https://search.datacite.org/works/10.15454/1.4861359557068474E12 after registration at https://urgi.versailles.inra.fr/Species/Vitis/Data-Sequences/Genotyping-data.

A genetic map was drawn using a homemade script in C to automate scaling and Gnuplot for graphical view (http://www.gnuplot.info/). Theoretical Illumina SNP positions were recalculated by re-mapping the flanking regions (2x60 bp) with NCBI/BLAST^®^ v2.2.31 (https://blast.ncbi.nlm.nih.gov/Blast.cgi) against the whole PN40024 reference genome sequence (assembly version 12X.V0 and 12X.V2, URGI: https://urgi.versailles.inra.fr/Species/Vitis/Data-Sequences/Genome-sequences. Update 27 October 2015).

### Phenotypic data

Phenotypic synthetic data, measured using the OIV [1] notation system were provided based on repository records. Sex, berry color, seeds, flavor, phenology, fertility, cluster and berry weight measurement data, as well as their respective OIV trait code number, are available in S7 Table.

### Data analysis

Basic statistics (SNPs frequency, distribution …) were calculated on the selected SNPs using GenomeStudio v2011.1 (Illumina Inc, San Diego, CA, USA). AMaCAID R program [74] was used to estimate the minimal number of markers needed to discriminate all genotypes.

First-degree parentage (i.e. parents-offspring trio and parent-offspring duo relationships) analysis and probability of identity (PI) were calculated using FAMOZ software [75]. Due to software limitation, SNP dataset for 783 cultivars was split in two subsets (1–5000 and 5001–10207) and PI was calculated on 8191 SNPs: SNPs designed on species, mapping on random or unknown chromosomes and with less than 5% minor allele frequencies were discarded.

Parentage results based on SNPs were compared to parentage analysis performed with 20 nSSRs available on 701 cultivars according to Lacombe et al. [16] and previous published relationships compiled in http://www.vivc.de/index.php. The confirmation of already known pedigrees enabled us to empirically determine a LOD score threshold value for validation. A small mismatch rate (mismatches / Number of loci < 0.0009) is considered consistent with the intrinsic error rate of the Illumina chip technology, so that individual pairs with less than 9 mismatches over >10000 SNPs are still considered potential parent-offspring pairs if all other SNPs and the LOD-score support this hypothesis.

Among first degree relationships, both parent-offspring duos and possible full-sib pairs were explored using the approach of Manichaikul et al. [76], based on the kinship (K) and the “zero identity-by-state” (IBS0) coefficients. A parent-offspring couple was retained if all their SNPs shared at least one allele (i.e. the identity-by-state “IBS0” coefficient is close to zero, except an error margin to account for genotyping errors, fixed at 0.001 for our dataset) and if the kinship (deflated by population structure) was within the class range for first degree relationships (0.177 < K < 0.354). We used the “KING-robust” method to account for the inflation bias in the kinship estimate due to population structure. KING-robust also allows exploring 2^nd^ and 3^rd^ degree relationships, however, given the existence in grape of several confounding factors (namely, the reiteration of some progenitors across generations and subpopulations), we used this option to generate a conservative list of unrelated cultivars, i.e. cultivars that have no parents within the dataset. In addition, KING-robust is based on different exclusion algorithm and initial assumptions than FAMOZ.

The genetic structure of the 783 cultivated grape samples was explored with the STRUCTURE software 2.3.4 [77]. In a preliminary analysis, we determined that the best model (p-value) was the one with independent allele frequencies, admixture and no geographic prior. Using this model, we explored the data with 10 independents runs for each K from 2 to 15. As in Bacilieri et al. [18], we used 5x10^4^ burn-in and 5x10^4^ samplings, as the analysis converged quickly. Indications of the most probable level of population subdivision were obtained using both Evanno’s delta-K [78] and the similarity among the ten independent runs. The results were summarized using the CLUMPP software 1.1.2 [79] and plotted with DISTRUCT 1.1 [80].

To refine the sample’s genetic structure, we also examined the data (783 individuals, 10207 SNPs) using the discriminant analysis of principal components (DAPC) implemented in the Adegenet package ver. 2.0.1 [81, 82] within R environment [83]. Prior clusters were identified by a sequential K-means clustering algorithm (find.clusters function) after data transformation by Principal Component Analysis (PCA). Then, a discriminant analysis (DA) used part of the principal components (PCs) to describe the clusters. K-means was ran with K varying from 1 to 20 and to ensure convergence we increased the number of starting points to 400 (default value = 10). The number of clusters was chosen based on the Bayesian Information Criterion (BIC) [47]. To avoid retaining too many dimensions at the DA step, we determined the optimal number of PCs using both “optim.a.score” and “xvalDapc” functions from Adegenet. The final cluster assignment was obtained after the DA step (posterior assignment of the DAPC analysis).

To assess the global differentiation and pairwise differentiation between clusters we used the R package mmod ver. 1.3.1 [84] using the classic Gst measure of Nei [85], the corrected measure G’st of Hedrick [86] and the D measure of Jost [87]. An exact G test was performed to obtain significance of the genotypic differentiation for each cluster pair using Genepop ver. 4.2 [88, 89]. The genetic distance of Rogers [90] between clusters was also calculated using the R package poppr ver. 2.2.0 [91, 92] and a NJ tree was constructed using the R package ape ver. 3.5 [93].

To further characterize the cultivars belonging to each cluster inferred from the DAPC analysis we built contingency tables for counts and percentages (R base package) using several cross-classifying factors available in the passport data (geographic origin, human usage) and the group assignment based on STRUCTURE analysis.

We used adegenet to calculate the genetic diversity (expected heterozygosity He) with the function Hs and to test differences in He between groups using the function Hs.test. Individual inbreeding coefficients were calculated using the—*het* option of the PLINK software (version 1.9, [94]). The departure from panmixia at the subpopulation (DAPC cluster) level was measured with the Fis coefficient (following [95]) and 100 bootstraps using the boot.ppfis function of the R package hierfstat (ver. 0.04–22 2015 [96]).

Linkage disequilibrium (LD) was explored using the *r*^*2*^_*VS*_ measure of Mangin et al. [97], correcting for both genetic structure and relatedness. The genetic structure matrix was obtained with the STRUCTURE method presented above. The relatedness matrix was obtained using PLINK (version 1.9, [94]). We focused our LD analysis on SNPs with a MAF > 5% using a sliding window covering 10 adjacent SNPs. These windows covered on average a chromosome length of 474 kbp (min = 81 kbp, max = 7633 kbp). The expected LD value was plotted as a non-linear function of physical distance according to the model of Hill and Weir [98]. LD extent was defined as the physical distance corresponding to a drop of the expected LD value below an arbitrary chosen level of *r*^*2*^_*VS*_ = 0.2. Haplotype non-recombining blocks as defined by Gabriel et al. [99], were calculated using the option—*blocks* in PLINK. The calculation was done both on the whole panel of 783 cultivars, and on each of the 8 subgroups resulting from the DAPC analysis. The resulting blocks were plotted on chromosomes using KaryoploteR R script [100].

Genome-wide association analysis (GWA) was carried out using the weighted-MLM model in Tassel (version 5.2.28, [101, 102]). The genetic structure matrix (used as a fixed-effect covariate) and relatedness matrix (used as a random effect) were obtained with the methods described above.

## Supporting information

S1 Table Passport data of 783 cultivars included in the study. (XLSX)

S2 Table List of parent-offspring trios. (XLSX)

S3 Table List of putative parentages detected with 20 nSSRs but not confirmed using 10207 SNPs. A—Parents-offspring trios invalidated. B—Parent-offspring duos invalidated.
(XLSX)

S4 Table List of parent-offspring pairs, validated by both FAMOZ and KING methods. A small mismatch rate (mismatches / Number of loci < 0.0009) is considered consistent with the intrinsic error rate of the Illumina chip technology, so that individual pairs with less than 9 mismatches over >10000 SNPs are still considered potential parent-offspring pairs if all other SNPs and the LOD-score support this hypothesis. The same reasoning applies to the IBS0 column.
(XLSX)

S5 Table List of cultivars with no parent relationships within the considered pool of 783 cultivars using the KING method. (XLSX)

S6 Table Detailed individuals characteristics: Geographical origin, usage, heterozygosity values (Fis), group assignations according to the DAPC and STRUCTURE at K = 4 and K = 8 analysis. (XLSX)

S7 Table Phenotypic data used for GWAs analysis. (XLSX)

S1 Fig Determination of the best K level of subdivision of the whole population according to its genetic structure (STRUCTURE software 2.3.4, Prichard et al. 2000), following the method of Evanno et al. [78]. H’ is the coefficient of similarity among runs, %runs stands for the number of runs that could be clustered under the same solution. The best K level in our analysis was 4, the last point before a drop of the stability of the model.
(PDF)

S2 Fig Representation of the rate of change of K (delta-K) according to population subdivision level (from 2 to 14), following the method of Evanno et al. [78]. (PDF)

S3 Fig Bayesian Information Criterion (BIC) according to the number of inferred clusters (K = 0–20). For this analysis 800 principal components were kept and the number of starting points was set at 400. The chosen number of clusters was K = 8.
(PDF)

S4 Fig Genetic relatedness between groups of grapevine cultivars. The genetic clusters were identified among 783 individuals by K-means and discriminant analysis of principal components (DAPC) based on 10207 SNPs. Scatter plots show a) 1–2 DA components, b) 1–3 DA components, c) 1–4 DA components, d) 2–3 DA components, e) 2–4 DA components and f) 3–4 DA components. The contribution percentages are 38.2%, 18.0%, 16.0% and 9.0% for the four first DA components.
(PDF)

S5 Fig Representation of contingency tables by square sizes to compare prior group assignments using K-means clustering a) the initial sample of cultivars with (783 genotypes) and without the offspring of each trios (- Kin, 665 genotypes) and b) the initial sample of cultivars (783 genotypes) without and with the adding (+ Geo) of additional cultivars from Georgia (data of De Lorenzis et al. [28]). (PDF)

S6 Fig Range of the linkage disequilibrium based on physical chromosomal distance at which average LD falls below 0.2 within each of the eight DAPC subgroups (number of individuals between brackets). (PDF)
