# Supplementary figures and images for: Extended diversity analysis of cultivated grapevine Vitis vinifera with 10K genome-wide SNPs
Source: PLoS One. 2018 Feb 8;13(2):e0192540. doi: 10.1371/journal.pone.0192540 (PMC5805323; doi:10.1371/journal.pone.0192540)

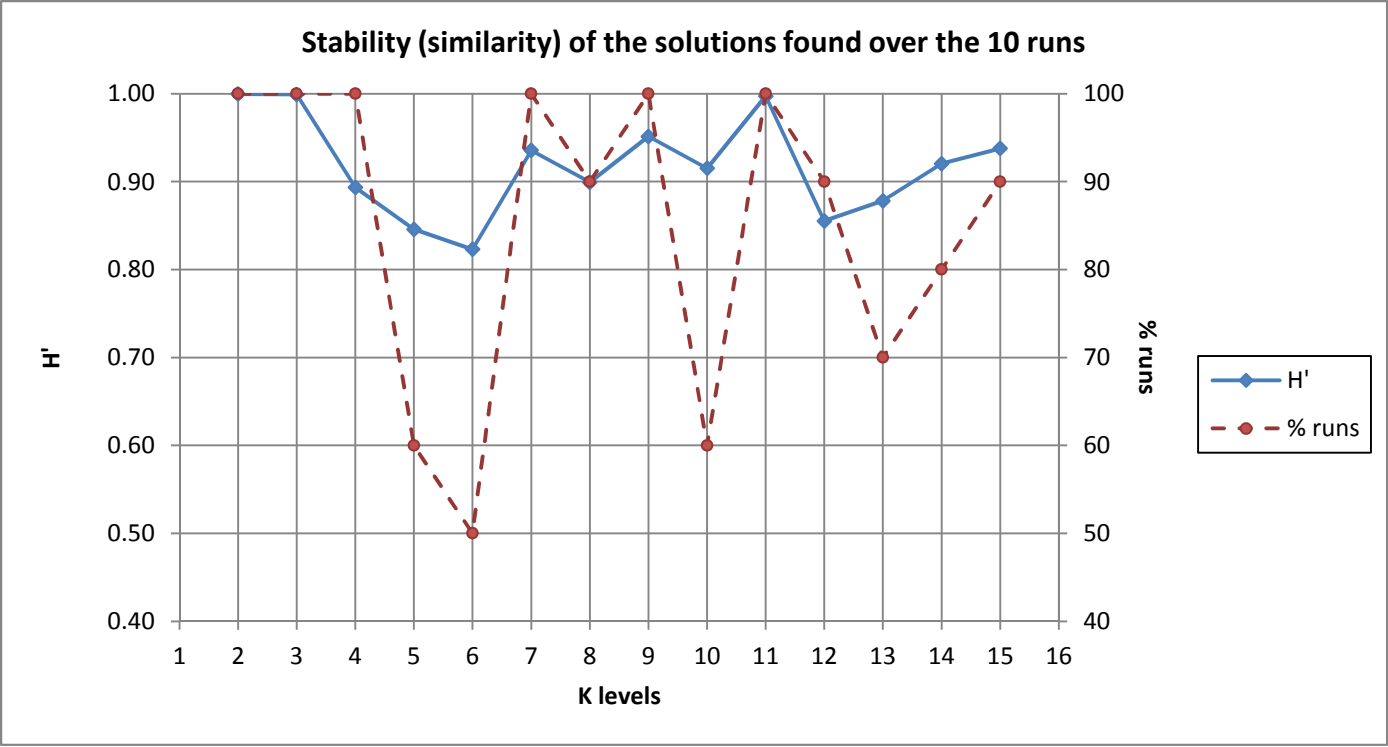

Supplement: S1 Fig — H’ is the coefficient of similarity among runs, %runs stands for the number of runs that could be clustered under the same solution. The best K level in our analysis was 4, the last point before a drop of the stability of the model. (PDF) [file pone.0192540.s008.pdf]

$$\text{DeltaK} = \text{mean}(|L''(K)|) / \text{sd}(L(K))$$

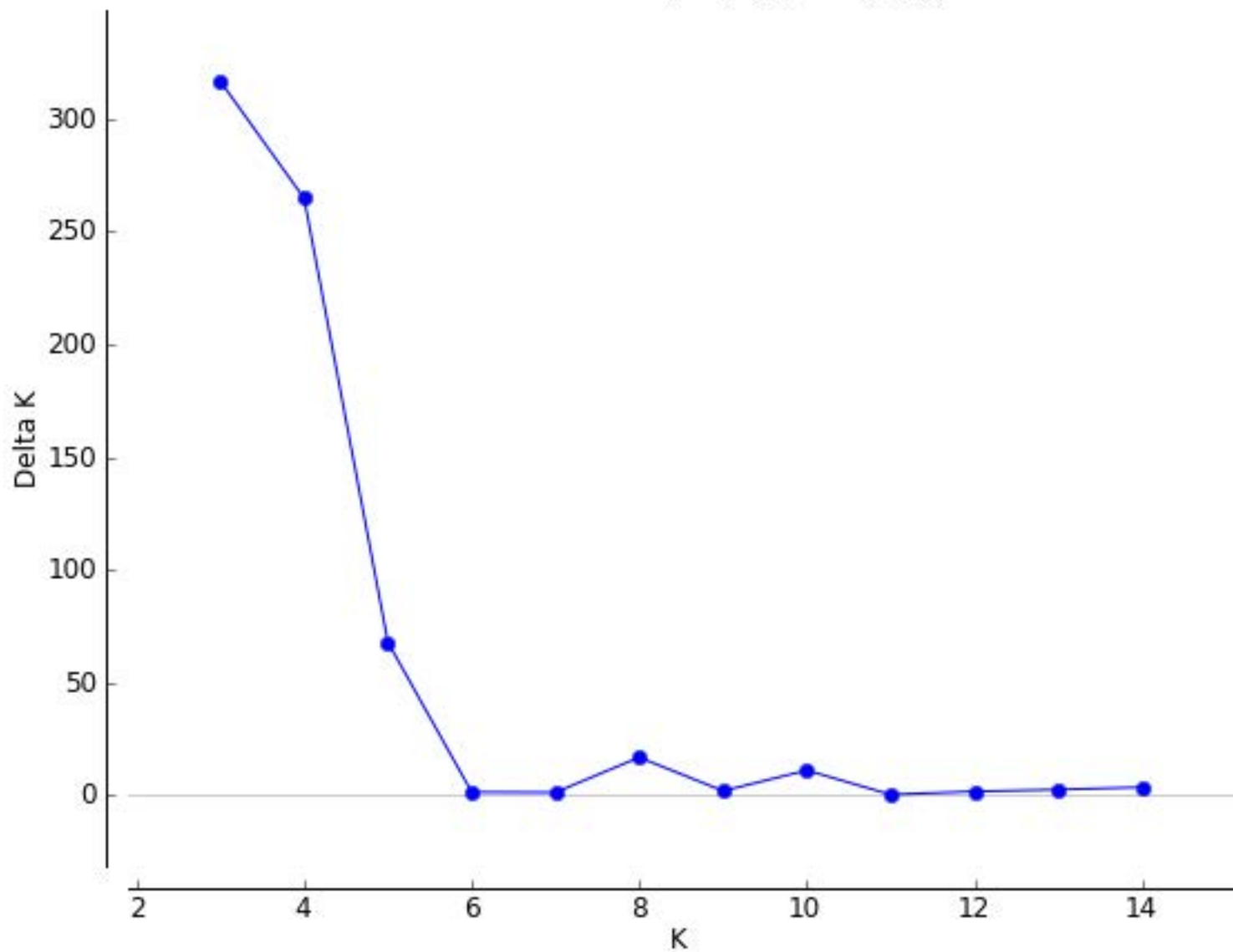

Supplement: S2 Fig — (PDF) [file pone.0192540.s009.pdf]

**Value of BIC vs number of clusters**

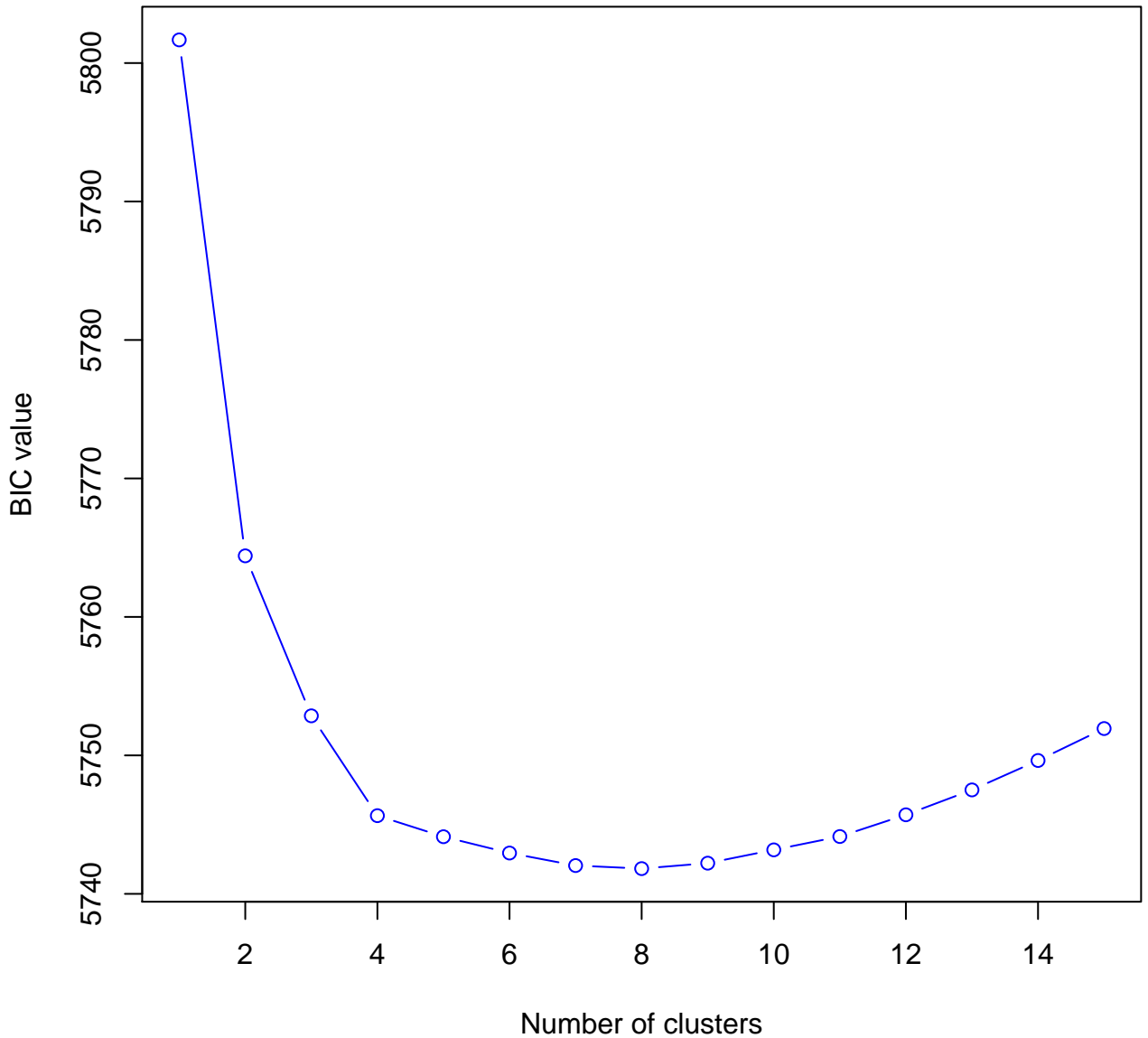

Supplement: S3 Fig — For this analysis 800 principal components were kept and the number of starting points was set at 400. The chosen number of clusters was K = 8. (PDF) [file pone.0192540.s010.pdf]

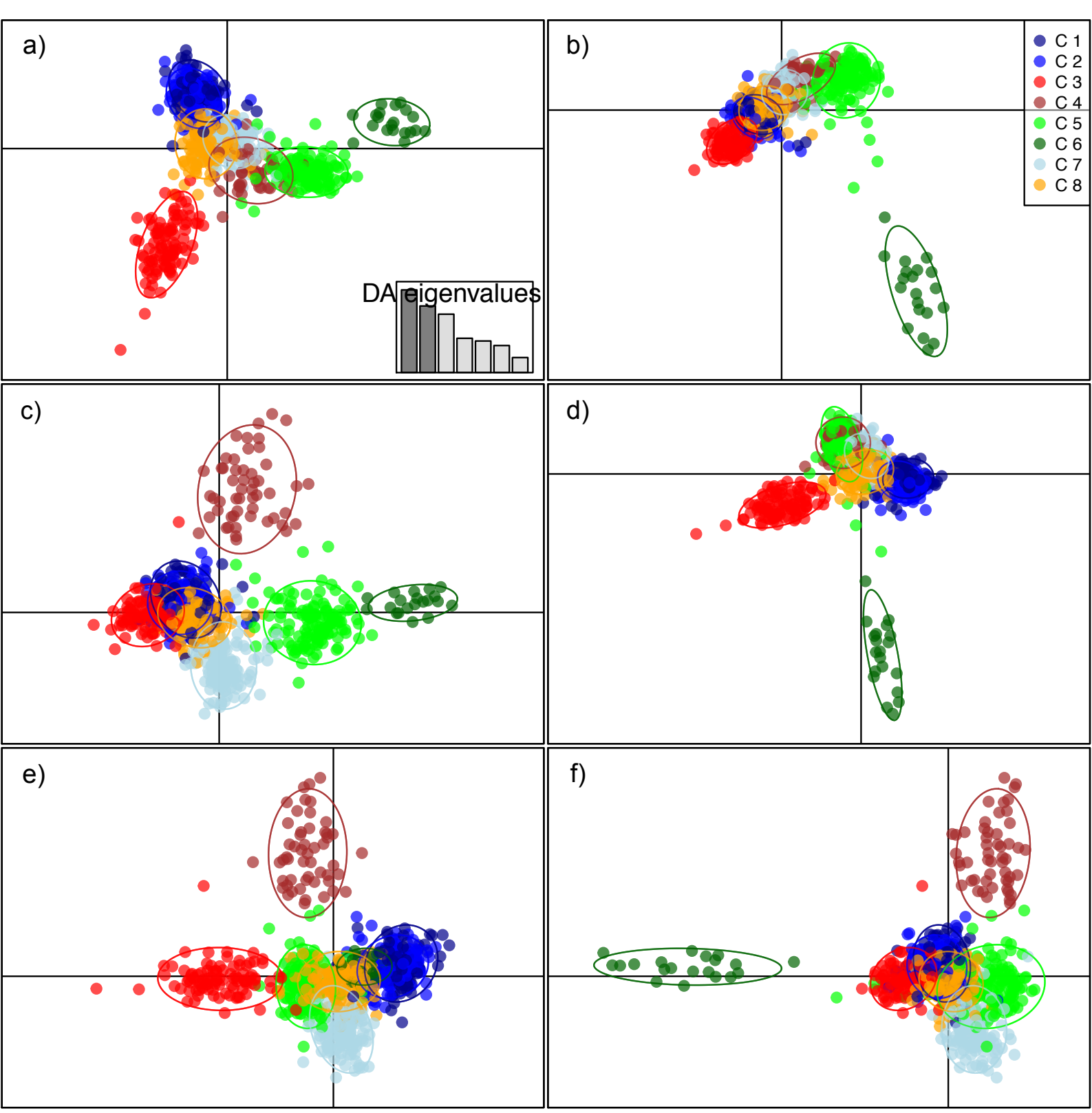

Supplement: S4 Fig — The genetic clusters were identified among 783 individuals by K-means and discriminant analysis of principal components (DAPC) based on 10207 SNPs. Scatter plots show a) 1–2 DA components, b) 1–3 DA components, c) 1–4 DA components, d) 2–3 DA components, e) 2–4 DA components and f) 3–4 DA components. The contribution percentages are 38.2%, 18.0%, 16.0% and 9.0% for the four first DA components. (PDF) [file pone.0192540.s011.pdf]

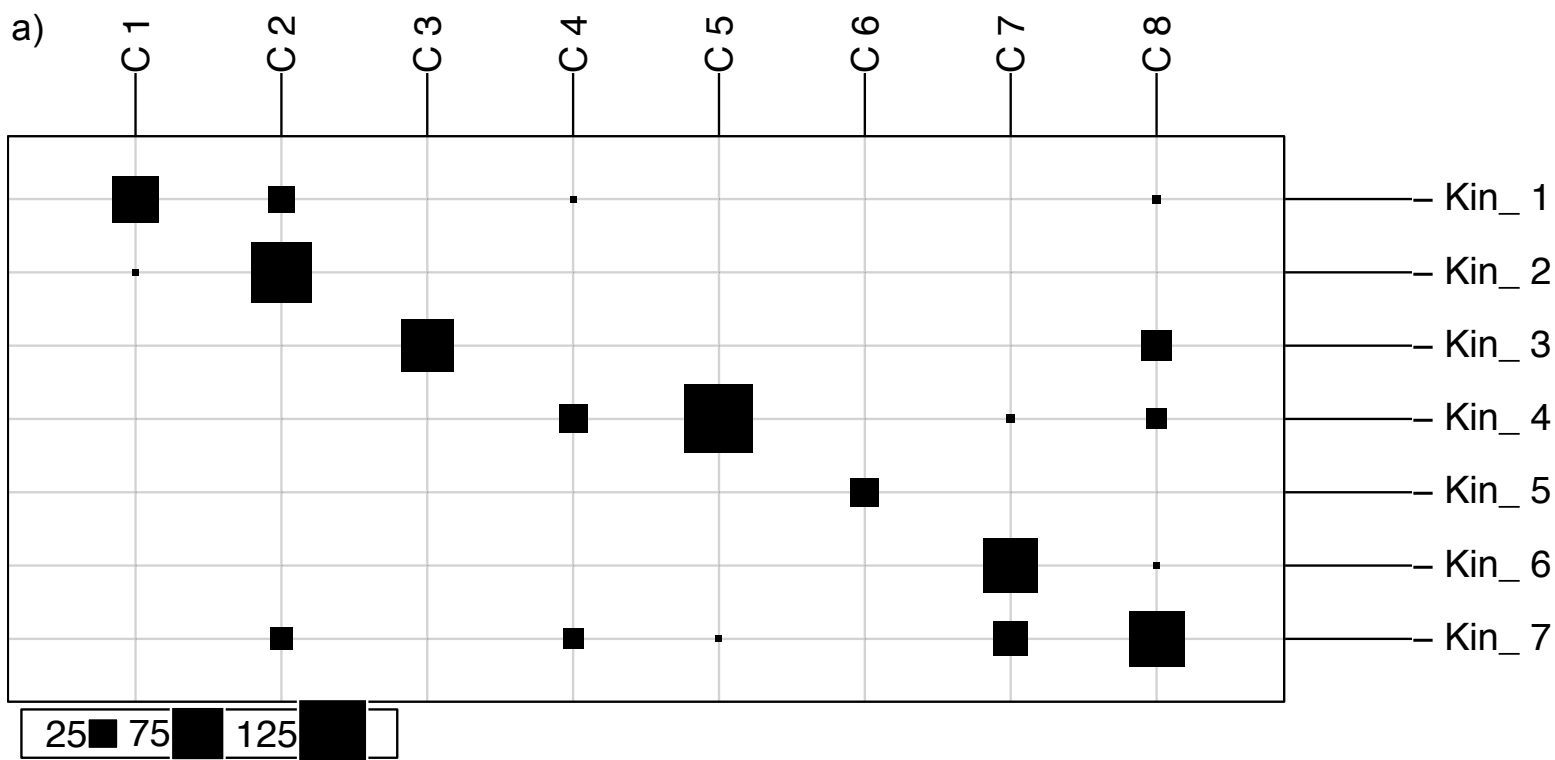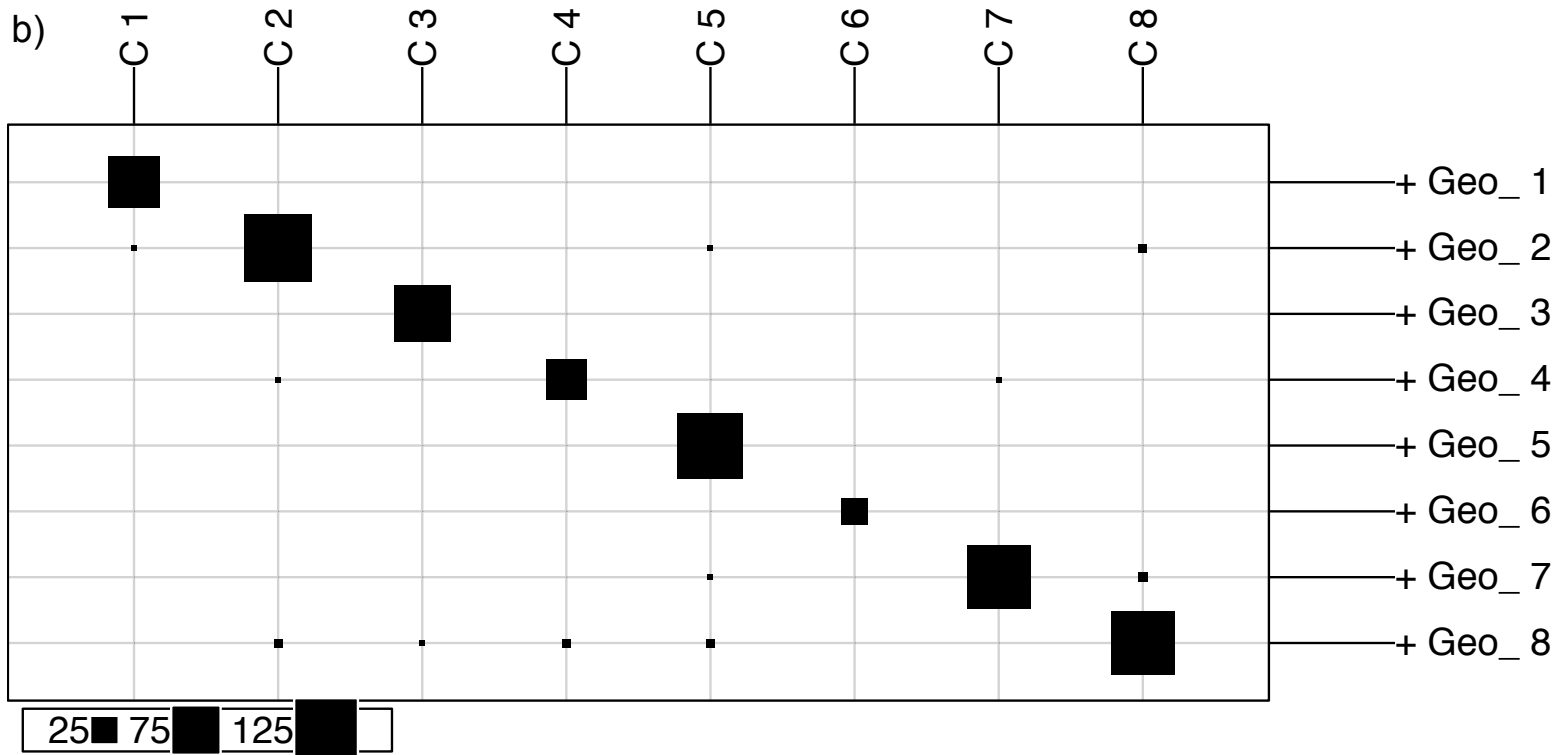

Supplement: S5 Fig — (PDF) [file pone.0192540.s012.pdf]

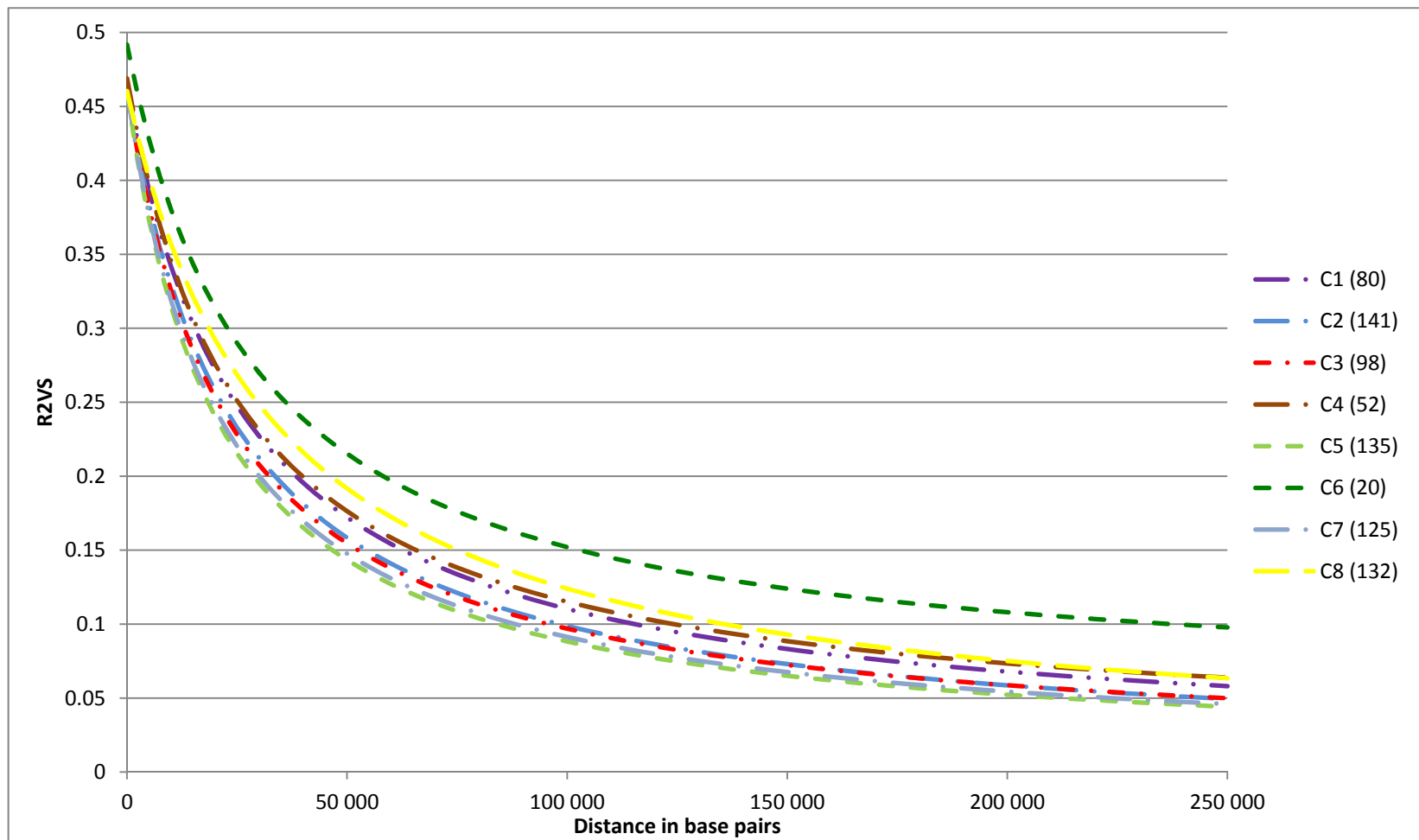

Supplement: S6 Fig — (PDF) [file pone.0192540.s013.pdf]
